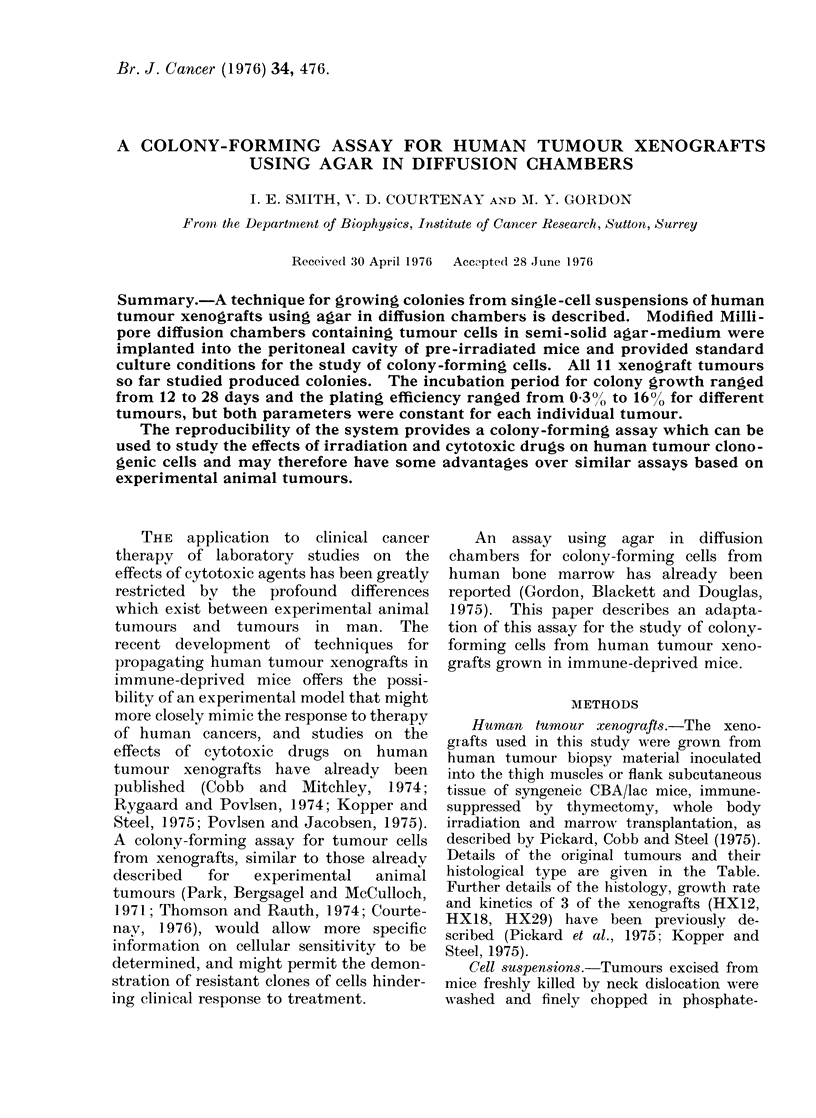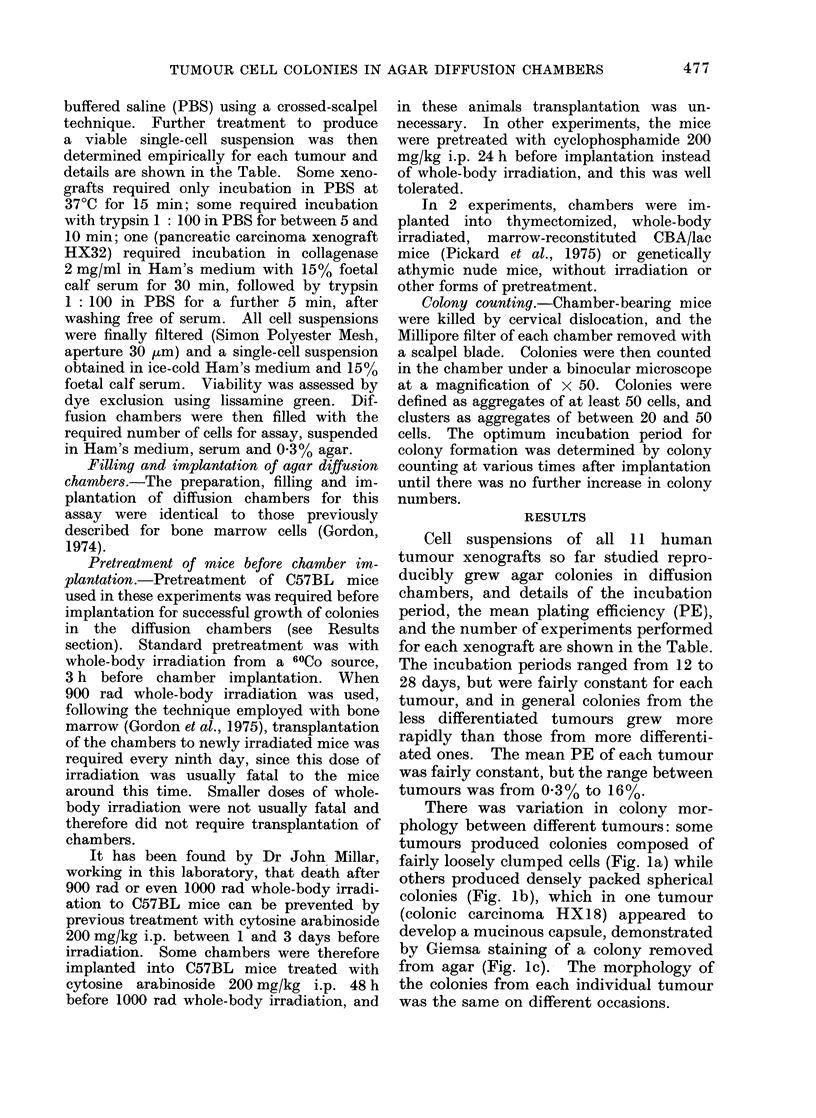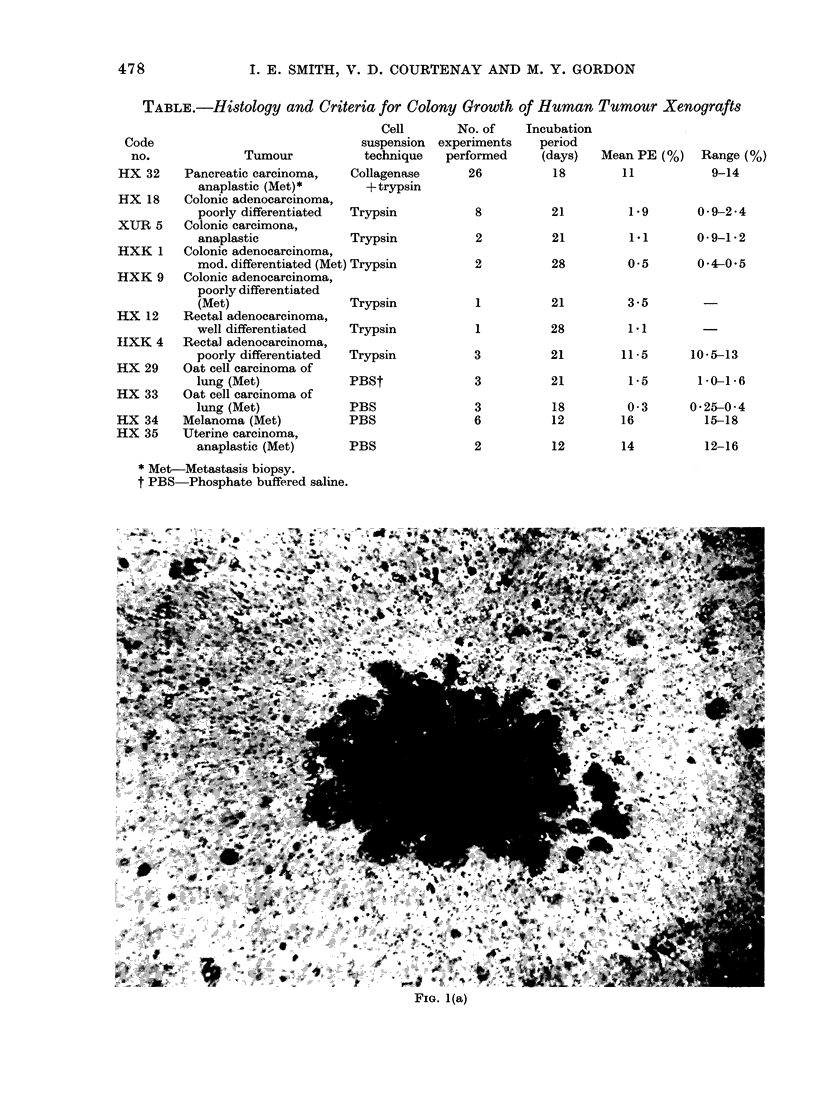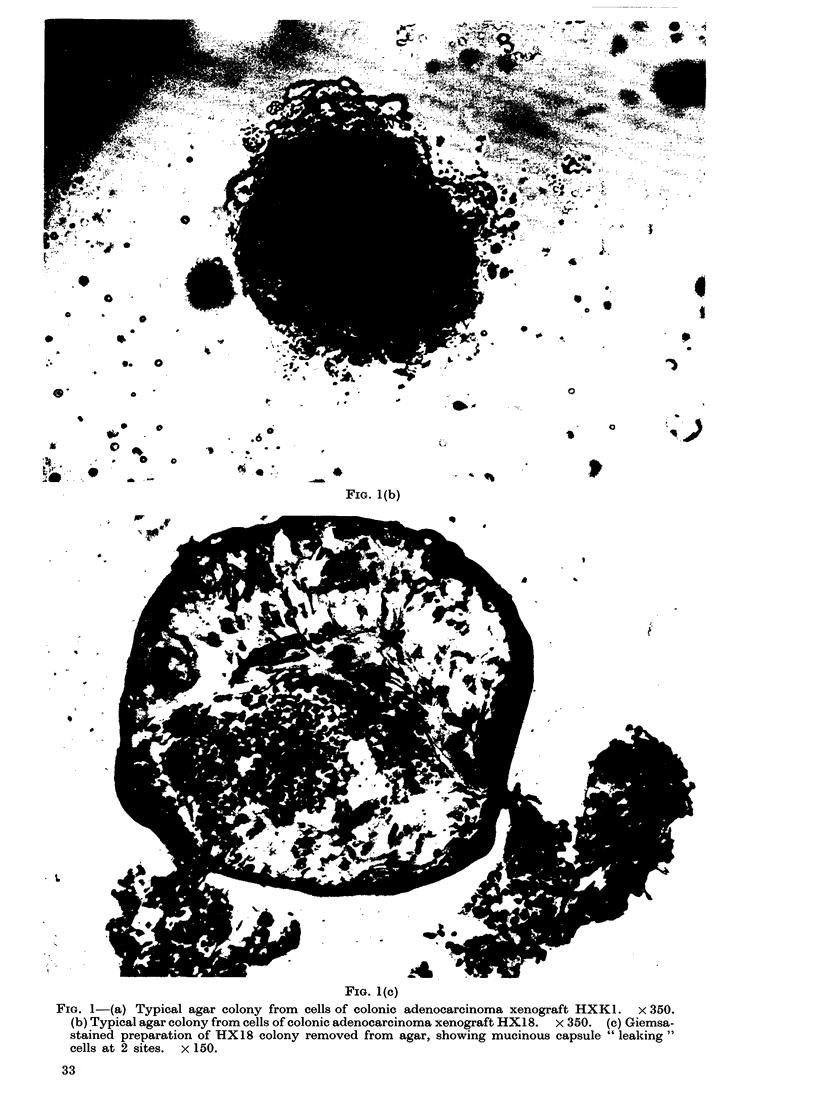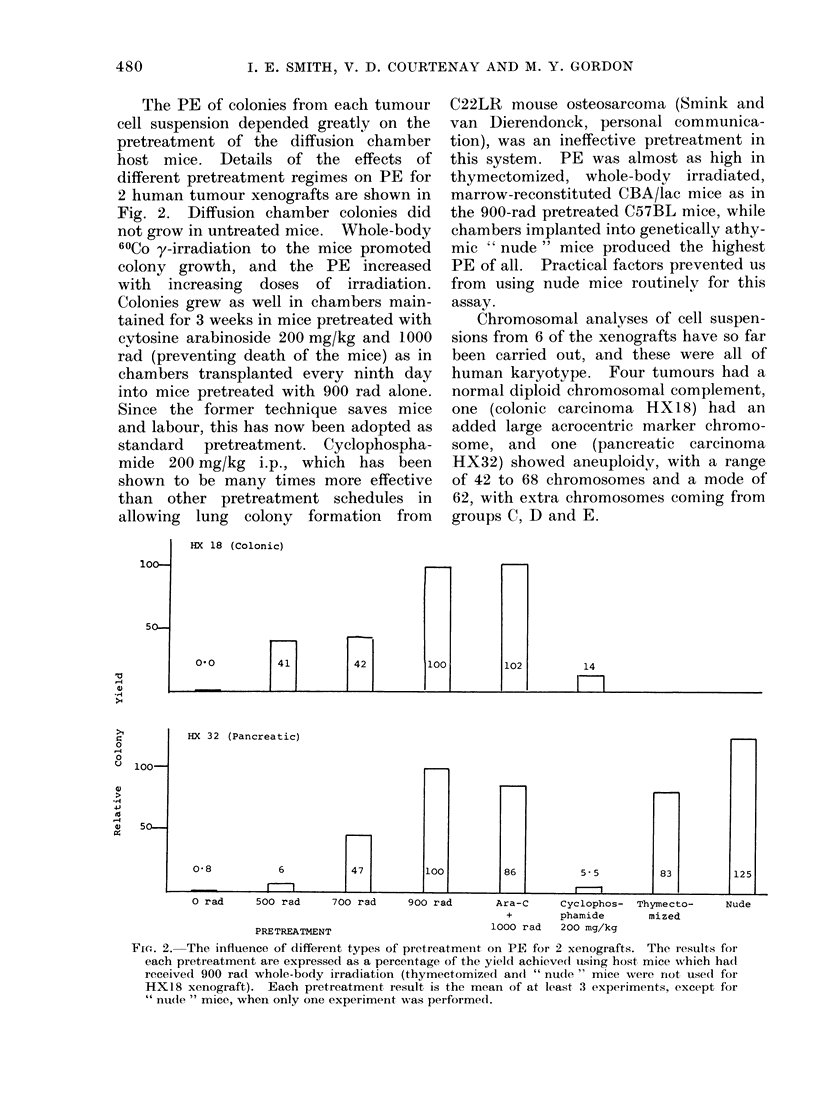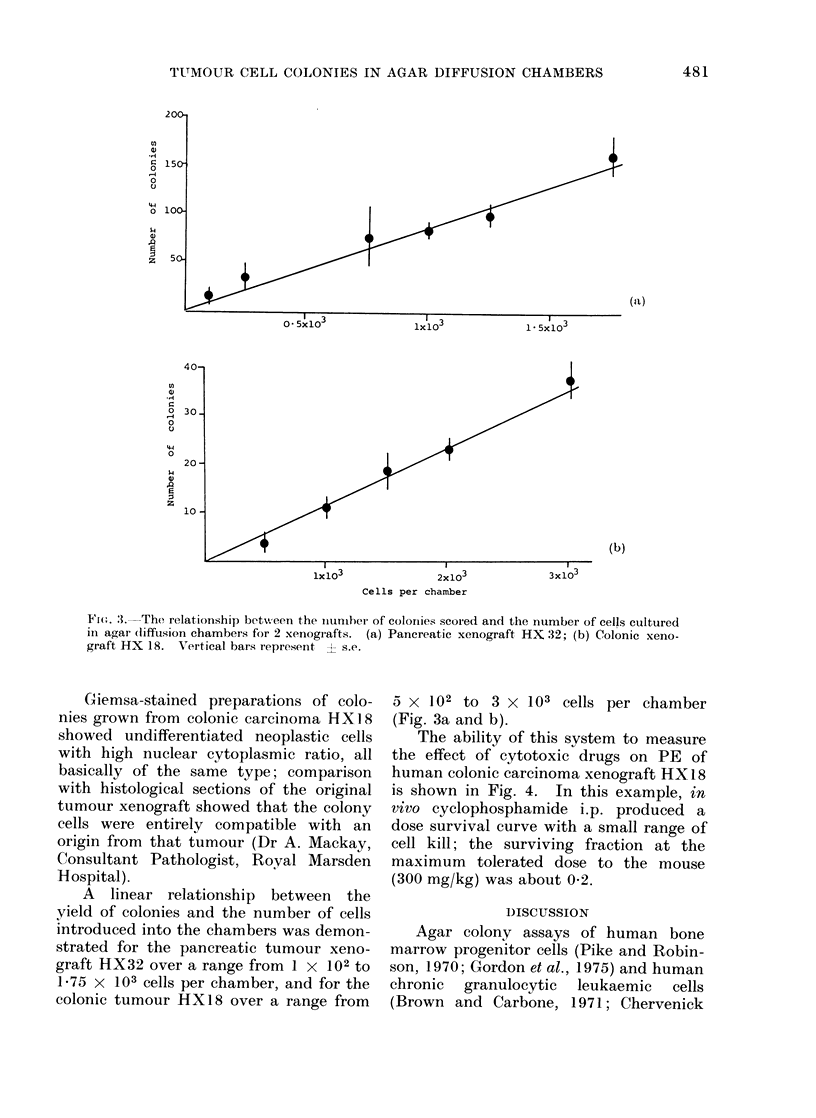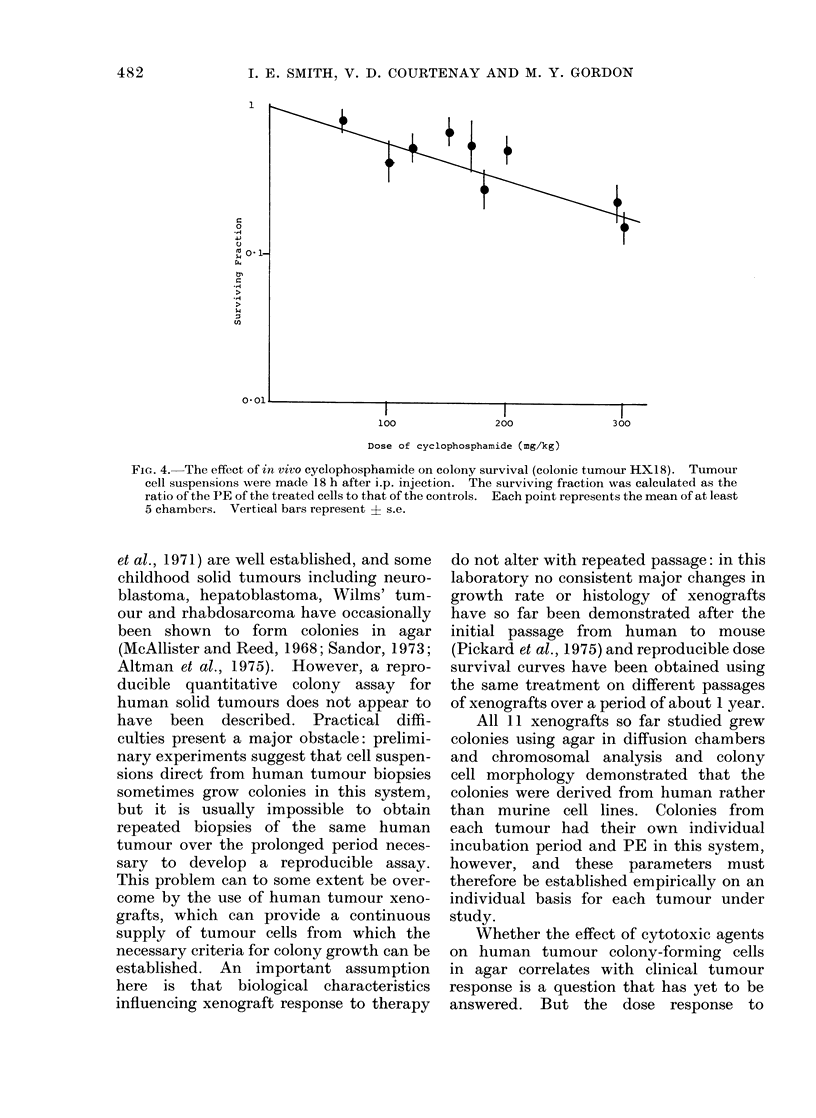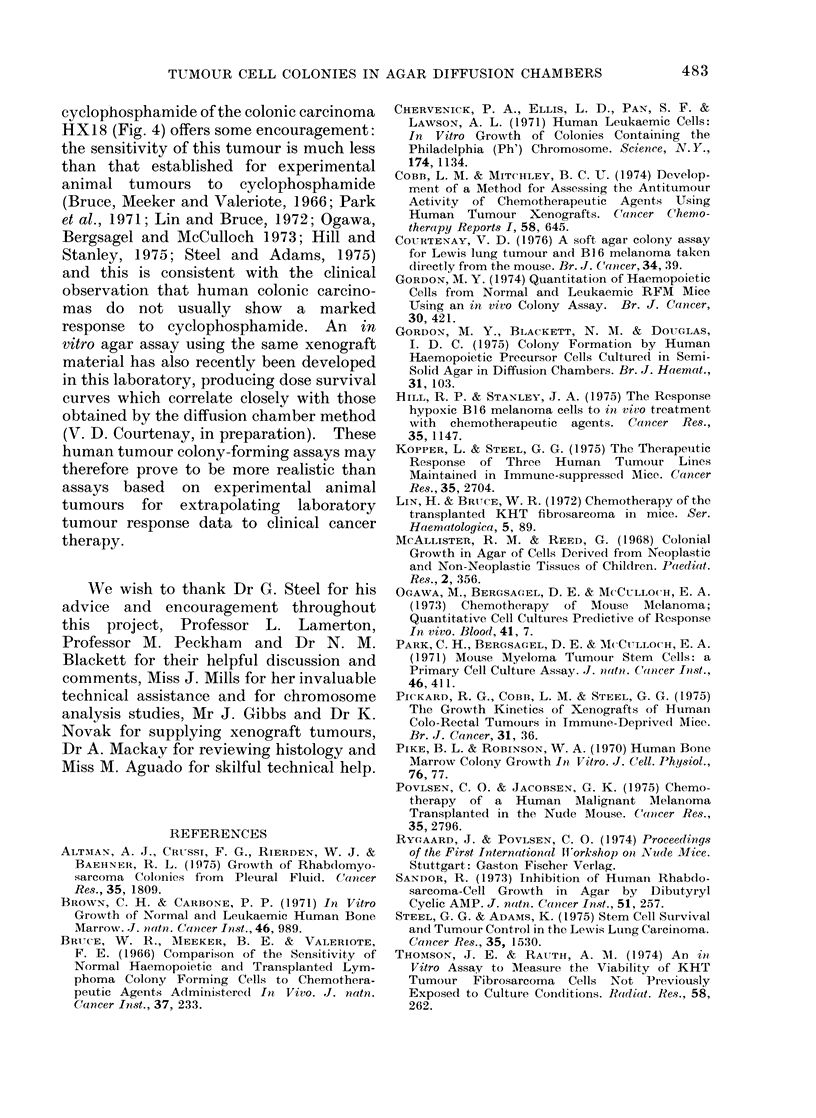# A colony-forming assay for human tumour xenografts using agar in diffusion chambers.

**DOI:** 10.1038/bjc.1976.201

**Published:** 1976-11

**Authors:** I. E. Smith, V. D. Courtenay, M. Y. Gordon

## Abstract

**Images:**


*Br. J. Cancer* (1976) **34**, 476.

### A COLONY-FORMING ASSAY FOR HUMAN TUMOUR XENOGRAFTS USING AGAR IN DIFFUSION CHAMBERS

I. E. SMITH, V. D. COURTENAY AND M. Y. GORDON

*From the Department of Biophysics, Institute of Cancer Research, Sutton, Surrey*

Received 30 April 1976 Accepted 28 June 1976

**Summary.—A technique for growing colonies from single-cell suspensions of human tumour xenografts using agar in diffusion chambers is described. Modified Millipore diffusion chambers containing tumour cells in semi-solid agar-medium were implanted into the peritoneal cavity of pre-irradiated mice and provided standard culture conditions for the study of colony-forming cells. All 11 xenograft tumours so far studied produced colonies. The incubation period for colony growth ranged from 12 to 28 days and the plating efficiency ranged from 0·3% to 16% for different tumours, but both parameters were constant for each individual tumour.**

**The reproducibility of the system provides a colony-forming assay which can be used to study the effects of irradiation and cytotoxic drugs on human tumour clonogenic cells and may therefore have some advantages over similar assays based on experimental animal tumours.**

THE application to clinical cancer therapy of laboratory studies on the effects of cytotoxic agents has been greatly restricted by the profound differences which exist between experimental animal tumours and tumours in man. The recent development of techniques for propagating human tumour xenografts in immune-deprived mice offers the possibility of an experimental model that might more closely mimic the response to therapy of human cancers, and studies on the effects of cytotoxic drugs on human tumour xenografts have already been published (Cobb and Mitchley, 1974; Rygaard and Povlsen, 1974; Kopper and Steel, 1975; Povlsen and Jacobsen, 1975). A colony-forming assay for tumour cells from xenografts, similar to those already described for experimental animal tumours (Park, Bergsagel and McCulloch, 1971; Thomson and Rauth, 1974; Courtenay, 1976), would allow more specific information on cellular sensitivity to be determined, and might permit the demonstration of resistant clones of cells hindering clinical response to treatment.

An assay using agar in diffusion chambers for colony-forming cells from human bone marrow has already been reported (Gordon, Blackett and Douglas, 1975). This paper describes an adaptation of this assay for the study of colony-forming cells from human tumour xenografts grown in immune-deprived mice.

### METHODS

*Human tumour xenografts.*—The xenografts used in this study were grown from human tumour biopsy material inoculated into the thigh muscles or flank subcutaneous tissue of syngeneic CBA/lac mice, immune-suppressed by thymectomy, whole body irradiation and marrow transplantation, as described by Pickard, Cobb and Steel (1975). Details of the original tumours and their histological type are given in the Table. Further details of the histology, growth rate and kinetics of 3 of the xenografts (HX12, HX18, HX29) have been previously described (Pickard *et al.*, 1975; Kopper and Steel, 1975).

*Cell suspensions.*—Tumours excised from mice freshly killed by neck dislocation were washed and finely chopped in phosphatebuffered saline (PBS) using a crossed-scalpel technique. Further treatment to produce a viable single-cell suspension was then determined empirically for each tumour and details are shown in the Table. Some xenografts required only incubation in PBS at 37°C for 15 min; some required incubation with trypsin 1 : 100 in PBS for between 5 and 10 min; one (pancreatic carcinoma xenograft HX32) required incubation in collagenase 2 mg/ml in Ham's medium with 15% foetal calf serum for 30 min, followed by trypsin 1 : 100 in PBS for a further 5 min, after washing free of serum. All cell suspensions were finally filtered (Simon Polyester Mesh, aperture 30 $\mu$m) and a single-cell suspension obtained in ice-cold Ham's medium and 15% foetal calf serum. Viability was assessed by dye exclusion using lissamine green. Diffusion chambers were then filled with the required number of cells for assay, suspended in Ham's medium, serum and 0·3% agar.

*Filling and implantation of agar diffusion chambers.*—The preparation, filling and implantation of diffusion chambers for this assay were identical to those previously described for bone marrow cells (Gordon, 1974).

*Pretreatment of mice before chamber implantation.*—Pretreatment of C57BL mice used in these experiments was required before implantation for successful growth of colonies in the diffusion chambers (see Results section). Standard pretreatment was with whole-body irradiation from a $^{60}$Co source, 3 h before chamber implantation. When 900 rad whole-body irradiation was used, following the technique employed with bone marrow (Gordon *et al.*, 1975), transplantation of the chambers to newly irradiated mice was required every ninth day, since this dose of irradiation was usually fatal to the mice around this time. Smaller doses of whole-body irradiation were not usually fatal and therefore did not require transplantation of chambers.

It has been found by Dr John Millar, working in this laboratory, that death after 900 rad or even 1000 rad whole-body irradiation to C57BL mice can be prevented by previous treatment with cytosine arabinoside 200 mg/kg i.p. between 1 and 3 days before irradiation. Some chambers were therefore implanted into C57BL mice treated with cytosine arabinoside 200 mg/kg i.p. 48 h before 1000 rad whole-body irradiation, and in these animals transplantation was unnecessary. In other experiments, the mice were pretreated with cyclophosphamide 200 mg/kg i.p. 24 h before implantation instead of whole-body irradiation, and this was well tolerated.

In 2 experiments, chambers were implanted into thymectomized, whole-body irradiated, marrow-reconstituted CBA/lac mice (Pickard *et al.*, 1975) or genetically athymic nude mice, without irradiation or other forms of pretreatment.

*Colony counting.*—Chamber-bearing mice were killed by cervical dislocation, and the Millipore filter of each chamber removed with a scalpel blade. Colonies were then counted in the chamber under a binocular microscope at a magnification of $\times$ 50. Colonies were defined as aggregates of at least 50 cells, and clusters as aggregates of between 20 and 50 cells. The optimum incubation period for colony formation was determined by colony counting at various times after implantation until there was no further increase in colony numbers.

### RESULTS

Cell suspensions of all 11 human tumour xenografts so far studied reproducibly grew agar colonies in diffusion chambers, and details of the incubation period, the mean plating efficiency (PE), and the number of experiments performed for each xenograft are shown in the Table. The incubation periods ranged from 12 to 28 days, but were fairly constant for each tumour, and in general colonies from the less differentiated tumours grew more rapidly than those from more differentiated ones. The mean PE of each tumour was fairly constant, but the range between tumours was from 0·3% to 16%.

There was variation in colony morphology between different tumours: some tumours produced colonies composed of fairly loosely clumped cells (Fig. 1a) while others produced densely packed spherical colonies (Fig. 1b), which in one tumour (colonic carcinoma HX18) appeared to develop a mucinous capsule, demonstrated by Giemsa staining of a colony removed from agar (Fig. 1c). The morphology of the colonies from each individual tumour was the same on different occasions.

TABLE.—*Histology and Criteria for Colony Growth of Human Tumour Xenografts*

| Code no. | Tumour | Cell suspension technique | No. of experiments performed | Incubation period (days) | Mean PE (%) | Range (%) |
|---|---|---|---|---|---|---|
| HX 32 | Pancreatic carcinoma, anaplastic (Met)* | Collagenase + trypsin | 26 | 18 | 11 | 9–14 |
| HX 18 | Colonic adenocarcinoma, poorly differentiated | Trypsin | 8 | 21 | 1·9 | 0·9–2·4 |
| XUR 5 | Colonic carcimona, anaplastic | Trypsin | 2 | 21 | 1·1 | 0·9–1·2 |
| HXK 1 | Colonic adenocarcinoma, mod. differentiated (Met) | Trypsin | 2 | 28 | 0·5 | 0·4–0·5 |
| HXK 9 | Colonic adenocarcinoma, poorly differentiated (Met) | Trypsin | 1 | 21 | 3·5 | — |
| HX 12 | Rectal adenocarcinoma, well differentiated | Trypsin | 1 | 28 | 1·1 | — |
| HXK 4 | Rectal adenocarcinoma, poorly differentiated | Trypsin | 3 | 21 | 11·5 | 10·5–13 |
| HX 29 | Oat cell carcinoma of lung (Met) | PBS† | 3 | 21 | 1·5 | 1·0–1·6 |
| HX 33 | Oat cell carcinoma of lung (Met) | PBS | 3 | 18 | 0·3 | 0·25–0·4 |
| HX 34 | Melanoma (Met) | PBS | 6 | 12 | 16 | 15–18 |
| HX 35 | Uterine carcinoma, anaplastic (Met) | PBS | 2 | 12 | 14 | 12–16 |

* Met—Metastasis biopsy.
† PBS—Phosphate buffered saline.

FIG. 1(a)

FIG. 1(b)

FIG. 1(c)

FIG. 1—(a) Typical agar colony from cells of colonic adenocarcinoma xenograft HXK1. ×350. (b) Typical agar colony from cells of colonic adenocarcinoma xenograft HX18. ×350. (c) Giemsa-stained preparation of HX18 colony removed from agar, showing mucinous capsule "leaking" cells at 2 sites. ×150.

The PE of colonies from each tumour cell suspension depended greatly on the pretreatment of the diffusion chamber host mice. Details of the effects of different pretreatment regimes on PE for 2 human tumour xenografts are shown in Fig. 2. Diffusion chamber colonies did not grow in untreated mice. Whole-body $^{60}Co$ $\gamma$-irradiation to the mice promoted colony growth, and the PE increased with increasing doses of irradiation. Colonies grew as well in chambers maintained for 3 weeks in mice pretreated with cytosine arabinoside 200 mg/kg and 1000 rad (preventing death of the mice) as in chambers transplanted every ninth day into mice pretreated with 900 rad alone. Since the former technique saves mice and labour, this has now been adopted as standard pretreatment. Cyclophosphamide 200 mg/kg i.p., which has been shown to be many times more effective than other pretreatment schedules in allowing lung colony formation from C22LR mouse osteosarcoma (Smink and van Dierendonck, personal communication), was an ineffective pretreatment in this system. PE was almost as high in thymectomized, whole-body irradiated, marrow-reconstituted CBA/lac mice as in the 900-rad pretreated C57BL mice, while chambers implanted into genetically athymic " nude " mice produced the highest PE of all. Practical factors prevented us from using nude mice routinely for this assay.

Chromosomal analyses of cell suspensions from 6 of the xenografts have so far been carried out, and these were all of human karyotype. Four tumours had a normal diploid chromosomal complement, one (colonic carcinoma HX18) had an added large acrocentric marker chromosome, and one (pancreatic carcinoma HX32) showed aneuploidy, with a range of 42 to 68 chromosomes and a mode of 62, with extra chromosomes coming from groups C, D and E.

FIG. 2.—The influence of different types of pretreatment on PE for 2 xenografts. The results for each pretreatment are expressed as a percentage of the yield achieved using host mice which had received 900 rad whole-body irradiation (thymectomized and " nude " mice were not used for HX18 xenograft). Each pretreatment result is the mean of at least 3 experiments, except for " nude " mice, when only one experiment was performed.

FIG. 3.—The relationship between the number of colonies scored and the number of cells cultured in agar diffusion chambers for 2 xenografts. (a) Pancreatic xenograft HX 32; (b) Colonic xenograft HX 18. Vertical bars represent ± s.e.

Giemsa-stained preparations of colonies grown from colonic carcinoma HX18 showed undifferentiated neoplastic cells with high nuclear cytoplasmic ratio, all basically of the same type; comparison with histological sections of the original tumour xenograft showed that the colony cells were entirely compatible with an origin from that tumour (Dr A. Mackay, Consultant Pathologist, Royal Marsden Hospital).

A linear relationship between the yield of colonies and the number of cells introduced into the chambers was demonstrated for the pancreatic tumour xenograft HX32 over a range from $1 \times 10^2$ to $1{\cdot}75 \times 10^3$ cells per chamber, and for the colonic tumour HX18 over a range from $5 \times 10^2$ to $3 \times 10^3$ cells per chamber (Fig. 3a and b).

The ability of this system to measure the effect of cytotoxic drugs on PE of human colonic carcinoma xenograft HX18 is shown in Fig. 4. In this example, *in vivo* cyclophosphamide i.p. produced a dose survival curve with a small range of cell kill; the surviving fraction at the maximum tolerated dose to the mouse (300 mg/kg) was about 0·2.

### DISCUSSION

Agar colony assays of human bone marrow progenitor cells (Pike and Robinson, 1970; Gordon *et al.*, 1975) and human chronic granulocytic leukaemic cells (Brown and Carbone, 1971; Chervenick

FIG. 4.—The effect of *in vivo* cyclophosphamide on colony survival (colonic tumour HX18). Tumour cell suspensions were made 18 h after i.p. injection. The surviving fraction was calculated as the ratio of the PE of the treated cells to that of the controls. Each point represents the mean of at least 5 chambers. Vertical bars represent ± s.e.

*et al.*, 1971) are well established, and some childhood solid tumours including neuroblastoma, hepatoblastoma, Wilms' tumour and rhabdosarcoma have occasionally been shown to form colonies in agar (McAllister and Reed, 1968; Sandor, 1973; Altman *et al.*, 1975). However, a reproducible quantitative colony assay for human solid tumours does not appear to have been described. Practical difficulties present a major obstacle: preliminary experiments suggest that cell suspensions direct from human tumour biopsies sometimes grow colonies in this system, but it is usually impossible to obtain repeated biopsies of the same human tumour over the prolonged period necessary to develop a reproducible assay. This problem can to some extent be overcome by the use of human tumour xenografts, which can provide a continuous supply of tumour cells from which the necessary criteria for colony growth can be established. An important assumption here is that biological characteristics influencing xenograft response to therapy do not alter with repeated passage: in this laboratory no consistent major changes in growth rate or histology of xenografts have so far been demonstrated after the initial passage from human to mouse (Pickard *et al.*, 1975) and reproducible dose survival curves have been obtained using the same treatment on different passages of xenografts over a period of about 1 year.

All 11 xenografts so far studied grew colonies using agar in diffusion chambers and chromosomal analysis and colony cell morphology demonstrated that the colonies were derived from human rather than murine cell lines. Colonies from each tumour had their own individual incubation period and PE in this system, however, and these parameters must therefore be established empirically on an individual basis for each tumour under study.

Whether the effect of cytotoxic agents on human tumour colony-forming cells in agar correlates with clinical tumour response is a question that has yet to be answered. But the dose response to cyclophosphamide of the colonic carcinoma HX18 (Fig. 4) offers some encouragement: the sensitivity of this tumour is much less than that established for experimental animal tumours to cyclophosphamide (Bruce, Meeker and Valeriote, 1966; Park *et al.*, 1971; Lin and Bruce, 1972; Ogawa, Bergsagel and McCulloch 1973; Hill and Stanley, 1975; Steel and Adams, 1975) and this is consistent with the clinical observation that human colonic carcinomas do not usually show a marked response to cyclophosphamide. An *in vitro* agar assay using the same xenograft material has also recently been developed in this laboratory, producing dose survival curves which correlate closely with those obtained by the diffusion chamber method (V. D. Courtenay, in preparation). These human tumour colony-forming assays may therefore prove to be more realistic than assays based on experimental animal tumours for extrapolating laboratory tumour response data to clinical cancer therapy.

We wish to thank Dr G. Steel for his advice and encouragement throughout this project, Professor L. Lamerton, Professor M. Peckham and Dr N. M. Blackett for their helpful discussion and comments, Miss J. Mills for her invaluable technical assistance and for chromosome analysis studies, Mr J. Gibbs and Dr K. Novak for supplying xenograft tumours, Dr A. Mackay for reviewing histology and Miss M. Aguado for skilful technical help.

### REFERENCES

ALTMAN, A. J., CRUSSI, F. G., RIERDEN, W. J. & BAEHNER, R. L. (1975) Growth of Rhabdomyosarcoma Colonies from Pleural Fluid. *Cancer Res.*, **35**, 1809.

BROWN, C. H. & CARBONE, P. P. (1971) *In Vitro* Growth of Normal and Leukaemic Human Bone Marrow. *J. natn. Cancer Inst.*, **46**, 989.

BRUCE, W. R., MEEKER, B. E. & VALERIOTE, F. E. (1966) Comparison of the Sensitivity of Normal Haemopoietic and Transplanted Lymphoma Colony Forming Cells to Chemotherapeutic Agents Administered *In Vivo*. *J. natn. Cancer Inst.*, **37**, 233.

CHERVENICK, P. A., ELLIS, L. D., PAN, S. F. & LAWSON, A. L. (1971) Human Leukaemic Cells: *In Vitro* Growth of Colonies Containing the Philadelphia (Ph') Chromosome. *Science, N.Y.*, **174**, 1134.

COBB, L. M. & MITCHLEY, B. C. U. (1974) Development of a Method for Assessing the Antitumour Activity of Chemotherapeutic Agents Using Human Tumour Xenografts. *Cancer Chemotherapy Reports I*, **58**, 645.

COURTENAY, V. D. (1976) A soft agar colony assay for Lewis lung tumour and B16 melanoma taken directly from the mouse. *Br. J. Cancer*, **34**, 39.

GORDON, M. Y. (1974) Quantitation of Haemopoietic Cells from Normal and Leukaemic RFM Mice Using an *in vivo* Colony Assay. *Br. J. Cancer*, **30**, 421.

GORDON, M. Y., BLACKETT, N. M. & DOUGLAS, I. D. C. (1975) Colony Formation by Human Haemopoietic Precursor Cells Cultured in Semi-Solid Agar in Diffusion Chambers. *Br. J. Haemat.*, **31**, 103.

HILL, R. P. & STANLEY, J. A. (1975) The Response hypoxic B16 melanoma cells to *in vivo* treatment with chemotherapeutic agents. *Cancer Res.*, **35**, 1147.

KOPPER, L. & STEEL, G. G. (1975) The Therapeutic Response of Three Human Tumour Lines Maintained in Immune-suppressed Mice. *Cancer Res.*, **35**, 2704.

LIN, H. & BRUCE, W. R. (1972) Chemotherapy of the transplanted KHT fibrosarcoma in mice. *Ser. Haematologica*, **5**, 89.

MCALLISTER, R. M. & REED, G. (1968) Colonial Growth in Agar of Cells Derived from Neoplastic and Non-Neoplastic Tissues of Children. *Paediat. Res.*, **2**, 356.

OGAWA, M., BERGSAGEL, D. E. & MCCULLOCH, E. A. (1973) Chemotherapy of Mouse Melanoma; Quantitative Cell Cultures Predictive of Response *In vivo*. *Blood*, **41**, 7.

PARK, C. H., BERGSAGEL, D. E. & MCCULLOCH, E. A. (1971) Mouse Myeloma Tumour Stem Cells: a Primary Cell Culture Assay. *J. natn. Cancer Inst.*, **46**, 411.

PICKARD, R. G., COBB, L. M. & STEEL, G. G. (1975) The Growth Kinetics of Xenografts of Human Colo-Rectal Tumours in Immune-Deprived Mice. *Br. J. Cancer*, **31**, 36.

PIKE, B. L. & ROBINSON, W. A. (1970) Human Bone Marrow Colony Growth *In Vitro*. *J. Cell. Physiol.*, **76**, 77.

POVLSEN, C. O. & JACOBSEN, G. K. (1975) Chemotherapy of a Human Malignant Melanoma Transplanted in the Nude Mouse. *Cancer Res.*, **35**, 2796.

RYGAARD, J. & POVLSEN, C. O. (1974) *Proceedings of the First International Workshop on Nude Mice.* Stuttgart: Gaston Fischer Verlag.

SANDOR, R. (1973) Inhibition of Human Rhabdosarcoma-Cell Growth in Agar by Dibutyryl Cyclic AMP. *J. natn. Cancer Inst.*, **51**, 257.

STEEL, G. G. & ADAMS, K. (1975) Stem Cell Survival and Tumour Control in the Lewis Lung Carcinoma. *Cancer Res.*, **35**, 1530.

THOMSON, J. E. & RAUTH, A. M. (1974) An *in Vitro* Assay to Measure the Viability of KHT Tumour Fibrosarcoma Cells Not Previously Exposed to Culture Conditions. *Radiat. Res.*, **58**, 262.